# Surface-bound iron: a metal ion buffer in the marine brown alga *Ectocarpus siliculosus*?

**DOI:** 10.1093/jxb/ert406

**Published:** 2013-12-24

**Authors:** Eric P. Miller, Lars H. Böttger, Aruna J. Weerasinghe, Alvin L. Crumbliss, Berthold F. Matzanke, Wolfram Meyer-Klaucke, Frithjof C. Küpper, Carl J. Carrano

**Affiliations:** ^1^Department of Chemistry and Biochemistry, San Diego State University, San Diego, CA 92182-1030, USA; ^2^Section Natural Sciences, Isotopes Laboratory, University of Lübeck, Ratzeburger Allee 160, D-23538 Lübeck, Germany; ^3^Department of Chemistry, Duke University, Durham, 27708-0346, NC, USA; ^4^European Molecular Biology Laboratory (EMBL), Hamburg Unit, c/o DESY, Notkestrasse 85, D-22607 Hamburg, Germany; ^5^Oceanlab, University of Aberdeen, Main Street, Newburgh AB41 6AA, Scotland, UK; * Present address: Department of Chemistry, Stanford University, Stanford, CA 94305-5080, USA.

**Keywords:** Apoplast, brown algae, cell wall, energy-dispersive X-ray analysis, histochemistry, iron, Mössbauer spectroscopy, surface binding, X-ray absorption.

## Abstract

Although the iron uptake and storage mechanisms of terrestrial/higher plants have been well studied, the corresponding systems in marine algae have received far less attention. Studies have shown that while some species of unicellular algae utilize unique mechanisms of iron uptake, many acquire iron through the same general mechanisms as higher plants. In contrast, the iron acquisition strategies of the multicellular macroalgae remain largely unknown. This is especially surprising since many of these organisms represent important ecological and evolutionary niches in the coastal marine environment. It has been well established in both laboratory and environmentally derived samples, that a large amount of iron can be ‘non-specifically’ adsorbed to the surface of marine algae. While this phenomenon is widely recognized and has prompted the development of experimental protocols to eliminate its contribution to iron uptake studies, its potential biological significance as a concentrated iron source for marine algae is only now being recognized. This study used an interdisciplinary array of techniques to explore the nature of the extensive and powerful iron binding on the surface of both laboratory and environmental samples of the marine brown alga *Ectocarpus siliculosus* and shows that some of this surface-bound iron is eventually internalized. It is proposed that the surface-binding properties of *E. siliculosus* allow it to function as a quasibiological metal ion ‘buffer’, allowing iron uptake under the widely varying external iron concentrations found in coastal marine environments.

## Introduction

Iron acquisition by marine organisms is of high interest due to its crucial role in many biological processes of the oceanic biota. In particular, algae have received considerable attention for their important ecological position as primary producers with high intracellular iron requirements related to photosynthesis, respiration, and electron transport. Satisfying these demands is complicated by the low solubility of Fe(III) in oxic seawater and the fact that a large fraction of this limited iron is complexed by strong natural organic chelates (Rue and Bruland, 1995). Evidenced by phytoplankton bloom formation through iron fertilization in high-nutrient, low-chlorophyll regions, iron is now well known to limit primary productivity in certain oceanic regimes (Martin and Fitzwater, 1988).

While the iron uptake and storage mechanisms of terrestrial/higher plants have been well studied, the corresponding systems in marine algae have received far less attention, with phytoplankton attracting most of this interest. Studies have shown that while some species of coccolithophores, diatoms, and unicellular green algae utilize unique mechanisms of iron uptake (Morrissey and Bowler, 2012), many acquire iron through the same general mechanisms as higher plants (Sutak *et al.*, 2012). The first of the two general mechanisms utilized by land plants (Strategy I) consists of a ferric chelate reductase in which transplasma membrane electron transport via flavohemoproteins reduces Fe(III) to Fe(II). Free ferrous iron is then taken up directly or reoxidized by a multicopper oxidase coupled to a high-affinity iron permease. Strategy II involves secretion by the plant cell, or associated bacteria, of high-affinity, iron-specific phytosiderophores. These phytosiderophores scavenge iron in the soil/extracellular milieu and are then transported back into the cell by sideroreceptors, after which iron dissociates (Morrissey and Bowler, 2012). However, while a phytosiderophore mechanism has never been confirmed in marine algae, it has been suggested to be present in the dinoflagellate *Prorocentrum minimum* (Trick *et al.*, 1983) and the coccolithophore *Emiliania huxleyi* (Boye and van den Berg, 2000). Having adapted to various iron-depleted environments, the cell-wall-containing unicellular eukaryote *Saccharomyces cerevisiae* has served as a model for investigations of these uptake strategies (Philpott, 2006). Genomic and iron uptake studies have confirmed the ability of yeast to exploit both of these strategies.

In contrast, the iron acquisition strategies of the multicellular macroalgae remain largely unknown. This is especially surprising since many of these organisms represent important ecological and evolutionary niches in the coastal marine environment. Together with the Stramenopile lineages of diatoms, golden-brown algae, and oomycetes, brown algae have evolved independently of other major photosynthetic lineages such as green plants and red algae and thus display many unique characteristics. Kelp forests and filamentous colonies dominate many coastal regions in terms of biomass and therefore play key roles in the natural product market (i.e. alginate and fucans), halogen biogeochemical cycle, and anthropogenic element remediation (Küpper *et al.*, 1998; Davis *et al.*, 2003; Raize *et al.*, 2004; Usov and Bilan, 2009; Benvegnu and Sassi, 2010). Indeed, brown algal biomass is so prevalent in some areas that certain industries view it as a nuisance, prompting considerations for brown algae as a feedstock for biofuels (Bringezu *et al.*, 2009; Vertès *et al*., 2010).

*Ectocarpus siliculosus* is a filamentous brown alga with a worldwide distribution along temperate coastlines, and is a nuisance as a ‘fouling’ organism on many man-made surfaces in the sea. It has some significant advantages as an experimental model and constitutes one of the best-studied seaweeds (Peters *et al.*, 2004; Charrier *et al.*, 2008). It can easily be cultivated in small volumes of seawater media both axenically and with associated bacteria, its entire lifecycle can be completed within a few months in culture (Müller *et al.*, 2008), and many molecular tools are available, including mutant collections, microarrays, and proteomics data. It has also recently become the first seaweed of which the entire genome has been sequenced and thus offers unprecedented opportunities for study (Cock *et al.*, 2010). Using a combination of genomic and spectroscopic approaches, this study group has recently shown that *E. siliculosus* acquires iron using a modified Strategy 1 reductive–oxidative mechanism, with iron storage likely involving a non-ferritin vacuole based system (Böttger *et al.*, 2012).

While it seems likely that the iron that many marine algae take up from the environment, irrespective of its detailed internalization mechanism, arrives at the cell surface by diffusion, there is growing evidence for more ‘active’ means of concentrating this element prior to uptake. It has been well established in both laboratory and environmentally derived samples that a large amount of iron can be ‘non-specifically’ adsorbed to the surface of marine algae (Hutchins *et al.*, 1999). This surface-bound iron may derive from simple electrostatic attraction between colloidal iron hydroxide particles and the cell surface, from *de novo* precipitation of iron hydroxo polymers from equilibrium solutions at the cell surface due to the increased surface pH relative to bulk seawater, or possibly from other mechanisms (Milligan *et al.*, 2009). While this surface adsorption phenomenon is widely recognized and has prompted the development of experimental protocols to eliminate its contribution to iron uptake studies, its potential exploitation as a concentrated iron source for marine algae is only now being recognized (Hudson and Morel, 1989; Tovar-Sanchez *et al.*, 2003). The extraordinary swarming behaviour of the related cyanobacterium *Trichodesmium* when confronted with particulate iron-containing dust is a spectacular example of active surface concentration of iron prior to actual uptake (Rubin *et al.*, 2011). More recent examples of possible surface concentration of iron come from the diatoms, the alveolate *Chromera velia,* and others where in some cases there is evidence that the surface-bound iron is ultimately internalized (Sutak *et al.*, 2010, 2012; Ingall *et al.*, 2013).

Since the cell wall of the brown algae is largely composed of anionic alginate polysaccharides, which account for 20–40% of their dry mass (Mabeau, 1987), it is scarcely surprising that the surface of these organisms should have considerable metal-binding properties. Sulphated polysaccharides are another structural polymer common to brown algae with metal-binding anionic functional groups. Indeed brown algae have been widely discussed for their potential in heavy metal biosorption applications (Davis *et al.*, 2003).

The current study explores the nature of the extensive and powerful iron binding on the surface of both laboratory and environmental samples of *E. siliculosus* and examines its potential effects on the subsequent uptake mechanism. It is proposed that the surface-binding properties of *E. siliculosus* function as a sort of metal ion ‘buffer’ allowing iron uptake under the widely varying external iron concentrations found in coastal marine environments.

## Materials and methods

### Biological material

For the initial XAS experiments at EMBL-DESY, *E. siliculosus* CCAP 1310/4—of which the genome has been sequenced recently (Cock *et al.*, 2010)—was obtained from the Culture Collection of Algae and Protozoa (CCAP) at the Scottish Association for Marine Science and grown axenically in modified Provasoli-enriched (Andersen, 2005) seawater from the Firth of Lorne (west coast of Scotland) at 14 °C under a 12/12 light/dark photocycle. For later experiments, *E. siliculosus* EcSil NZ KU 1–3♂ (CCAP 1310/56) was grown axenically in modified Provasoli-enriched Scripps Pier seawater (SPSW) at 17 °C under a 12/12 light/dark photocycle. The iron content of SPSW was determined to be approximately 4nM and is thus defined as the concentration for iron-limited growth. Prior to all experiments*, Ectocarpus* was starved for a period of 5–10 days under iron-limited conditions. Iron-replete conditions were obtained by adding Fe-EDTA to SPSW at 30 μM. Environmental samples of *E. siliculosus* were harvested from the shore near Dunstaffnage Marine Lab in Oban, Scotland.

### Surface-bound iron studies

^55^FeCl_3_ radionuclide was obtained from Perkin-Elmer and used to prepare the ^55^FeEDTA solution used as an iron source. Internalized iron was determined after all surface-bound Fe(III) species were removed from *E. siliculosus* using the titanium(III)-citrate/EDTA procedure as described by Hudson and Morel (1989). Total iron taken up (both external and internal) was determined using samples not washed with the Ti-citrate EDTA reagent. Surface-bound iron was defined as the ^55^Fe signal of cells not treated with Ti less the internalized iron signal of titanium washed replicates. For pulse-chase experiments, *E. siliculosus* cells were incubated in 100ml of 30 μM radiolabled ^55^FeEDTA SPSW medium for 6h. Cells were then removed from the medium, washed with 5ml low iron SPSW (~4nM ^56^Fe), and resuspended in 100ml of either (a) ultra-low iron oligotrophic Pacific Ocean water (~0.1nM ^56^Fe) or (b) SPSW containing 30 μM ‘cold’ ^56^FeEDTA. All samples were filtered on 5 μm Millipore polycarbonate filters and washed with 25ml of artificial sea water (Amin *et al.*, 2007). Thorough washing with both the titanium reagent and subsequently with artificial seawater is essential to eliminate artefacts caused by surface binding of Fe(III) and Fe(II), respectively. Filtered cells were dried under a vacuum and placed in scintillation vials with 1ml sodium hypochlorite to bleach the chlorophyll and reduce quenching effects. Vials were then heated in a 55 °C water bath for 1h and allowed to vent overnight at room temperature to allow chloride evaporation. Hionic Fluor liquid scintillation fluid (15ml; Perkin-Elmer) was added to each of the vials which were incubated in the dark for at least 2h to eliminate any background chemiluminesence. The ^55^Fe taken up was measured on a Beckman-Coulter LS 6500 scintillation counter using the tritium channel. Total iron uptake per mg dry *E. siliculosus* cells was calculated based on specific activity, measured count rates, scintillation counting efficiency, and biomass measurements.

### Binding constant and equilibrium studies

Cell-surface iron affinity is expressed as the equilibrium constant *K′*_*eff*_:

$$\text{Cell}+{\left[{\text{Fe}}_{\text{T}}^{3+}\right]}_{\text{eq}}⇆\text{FeCell }K\;{'}_{eff}$$ (reaction 1)

${\text{Fe}}_{\text{T}}^{3+}$ in reaction 1 (also known as Fe(III)’) represents the total soluble and unbound aqueous iron(III) in all of its hydrolysed forms (under these conditions: Fe(H_2_O)_6_^3+^ + Fe(H_2_O)_5_(OH)^2+^ + Fe(H_2_O)_4_(OH)_2_^+^ + Fe_2_(H_2_O)_8_(OH)_2_^4+^). Cell-surface iron affinity constants were estimated by measuring surface-bound iron (reaction 2) with varying concentrations of EDTA and varying pH while [Fe(III)] remained fixed at 30 μM:

Cell+FeEDTA'⇆FeCell+EDTA'       K ' (reaction 2)

where FeEDTA' represents the total EDTA/iron(III) species (FeEDTA^–^ + FeHEDTA + Fe(OH)EDTA^2–^) and EDTA' represents the total EDTA species (EDTA^4–^ + HEDTA^3–^ + H_2_EDTA^2–^ + H_3_EDTA^1–^ + H_4_EDTA + H_5_EDTA^1+^) in equilibrium under these conditions. In the calculations, it was assumed that only the hydrated iron(III) species (i.e. Fe(H_2_O)_6_^3+^ + Fe(H_2_O)_5_(OH)^2+^ + Fe(H_2_O)_4_(OH)_2_^+^ + Fe_2_(H_2_O)_8_(OH)_2_^4+^) bind to the cell surface while neither EDTA' nor FeEDTA' do. Relatively constant values (± 0.5 log unit) were calculated for ${\text{Fe}}_{\text{T}}^{3+}$ at a constant pH over a range of experimental conditions (10–18mg cells exposed to 33–1500 μM EDTA in the presence of 30 μM Fe(III) at pH 8.7±0.1), which confirms the validity of the assumptions and the computational approach. Details of the data analysis can be found in the Supplementary Material S1 (available at *JXB* online).

### Histochemistry

*E. siliculosus* was grown under iron-replete conditions (30 μM Fe(III)/300 μM EDTA) prior to fixation, dehydration, and embedding. Cells were fixated in a 0.1M phosphate buffer solution containing 2% (w/v) paraformaldehyde, 1% (w/v) glutaraldehyde, and 1% (w/v) caffeine for 2h. The fixed cells were then washed with 0.1M phosphate buffer and dehydrated in successive ethanol baths of 30, 50, 75, 85, 95, and 100% (×3). The cells were then embedded in 1:1 (v/v) ethanol/LR White resin (LWR; EMS, Hatfield, PA, USA) for 3h followed by 100% LWR overnight in gelatin capsules under vacuum. Sections (3 μm) were cut on a Reichert-Jung Ultracut E microtome and deposited on glass slides. Perls staining and 3,3-diaminobenzidine (DAB) intensification procedure was performed as previously described (Meguro *et al.*, 2007; Roschzttardtz *et al.*, 2009). Briefly, sections were incubated on glass slides with equal volumes 4% (v/v) HCl and 4% potassium ferrocyanide (Perls staining solution) for 45 minutes. After washing with distilled H_2_O, sections were incubated in a methanol solution containing 0.01M NaN_3_ and 0.3% (v/v) H_2_O_2_ for 1h and then washed with 0.1M phosphate buffer. DAB intensification was achieved by incubating sections in a 0.1M phosphate buffer solution containing 0.00025–0.005% (w/v) DAB (Sigma), 0.005% (v/v) H_2_O_2_, and 0.005% (w/v) CoCl_2_ for 30 minutes. The sections were then washed with H_2_O before imaging with a Leica DMRB microscope.

### Energy-dispersive X-ray analysis

Cells were fixed in a 0.1M phosphate buffer solution containing 2% (w/v) paraformaldehyde, 1% (w/v) glutaraldehyde, and 1% (w/v) caffeine for 2h. The fixed cells were then washed with 0.1M phosphate buffer and dehydrated in successive ethanol baths of 30, 50, 75, 85, 95, and 100% (×3). The cells were then embedded in 1:1 (v/v) ethanol/LWR for 3h followed by 100% LWR overnight in gelatin capsules under vacuum. Sections (3 μm) were cut on a Leica EMUC6 microtome and deposited on glass slides. Slides were coated with platinum in a Quorum Technologies Q150T ES sputter coater. Platinum-coated samples were analysed under high vacuum in a Quanta 450 FEG environmental scanning electron microscope equipped with an Oxford Instruments INCA energy-dispersive X-ray microanalysis system.

### Transmission Mössbauer spectroscopy

For transmission Mössbauer spectroscopy (TMS), algal samples were either washed with the titanium citrate/EDTA reagent in order to remove adventitious iron from the algal surface, or they were used untreated. Cells were harvested by vacuum-assisted filtration. Pellets were weighed, transferred into Delrin Mössbauer sample holders, frozen in liquid nitrogen, and kept at this temperature until measurement except for overnight transport on dry ice. The Mössbauer spectra were recorded in the horizontal transmission geometry using a constant acceleration spectrometer operated in conjunction with a 512-channel analyser in the time-scale mode. The detector consisted of a proportional counter filled with argon/methane (9:1). The source was at room temperature and consisted of 1.4 GBq [^57^Co] diffused in Rh foil (WissEl, Starnberg, Germany). The spectrometer was calibrated against α-iron at room temperature. For measurements at 77 K, samples were placed in a continuous-flow cryostat (Oxford Instruments). For measurements at 4.3K and 1.8 K, a helium bath cryostat (MD306, Oxford Instruments) was employed. Spectral data were transferred from the multichannel analyser to a PC for further analysis employing the public domain Vinda program on an Excel 2003 platform. Isomer shift δ, quadrupole splitting ΔEQ, magnetic hyperfine splitting B_hf_, line width Γ, and percentage of the total absorption area were obtained by least-squares fits of Lorentzian lines to the experimental spectra. All values are rounded to the last given digit. The isomers shifts, the quadrupole splitting, and the line width are given in mm s^–1^. Hyperfine fields are given in Tesla. The relative area is given in parts per hundreds.

### X-ray absorption spectroscopy

For X-ray absorption spectroscopy (XAS), the K-edge iron X-ray absorption spectrum was recorded at the beamline D2 of the EMBL Outstation Hamburg at DESY, Germany. The DORIS storage ring operated at 4.5 GeV with the positron beam current ranging from 145 to 80 mA. A Si(111) double-crystal monochromator scanned X-ray energies around Fe K-edge (6.9–7.85 KeV). Harmonic rejection was achieved by a focusing mirror (cut-off energy at 20.5 KeV) and a monochromator detuning to 50% of its peak intensity. The sample cells were mounted in a two-stage Displex cryostat and kept at about 20 K. The X-ray absorption spectra were recorded as Fe K_α_ fluorescence spectra with a Canberra 13-element Ge solid-state detector. Data reduction, such as background removal, normalization, and extraction of the fine structure, was performed with KEMP2 (Korbas *et al.*, 2006; Wellenreuther and Meyer-Klaucke, 2009) assuming a threshold energy E_0,Fe_ = 7120eV. Sample integrity during exposure to synchrotron radiation was checked by monitoring the position and shape of the absorption edge on sequential scans. No changes were detectable.

### Extended X-ray analysis of fine structure

For extended X-ray analysis of fine structure (EXAFS), a forward Fourier transformation was performed for a k-range from 2.1 to 10.0 Å^–1^ using a Hanning window function. The final overall fit of the model (*vide infra)* uses only two-legged scattering paths of Fe-O and Fe-[O]-C. The fits were performed in R-space using a Hanning-type window with r from 1 to 3.4 Å. For the least-square fits in R-space, a k-weight of 2 was used. All corrections and fits were performed by the Demeter 0.9.13 program package of Ravel and Newville (2005).

## Results

### Kinetics and thermodynamics of surface-bound iron

**Fig. 1.**
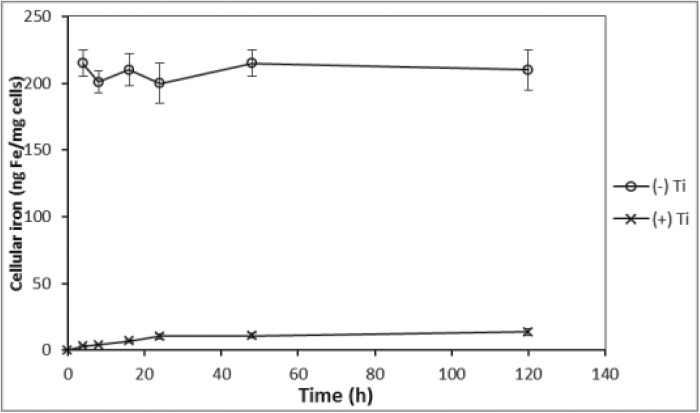
Uptake of ^55^Fe by *Ectocarpus* exposed to 30 μM ^55^FeEDTA as a function of time in samples washed or not washed with Ti-citrate-EDTA reagent. Data are mean ± standard deviation of triplicate measurements. Other conditions as described in the text.

**Fig. 2.**
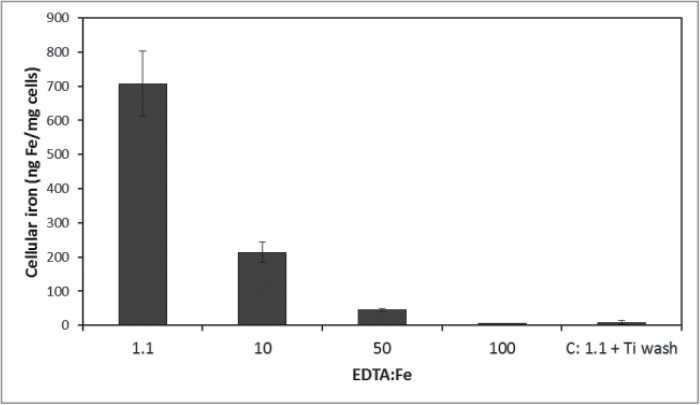
Cellular surface-bound ^55^Fe (i.e. sample not washed with Ti-citrate-EDTA reagent) as a function of excess EDTA in the media containing 30 μM ^55^FeEDTA. Error bars are mean ± standard deviation of triplicate measurements. Other conditions as described in the text.

Previous studies indicated that iron is internalized by *Ectocarpus* from FeEDTA in a time- and concentration-dependent manner (Böttger *et al.*, 2012) with the soluble Fe(III) species known as Fe(III)′ as the likely biological substrate as opposed to the intact Fe(III)-EDTA complex. Internal iron was determinable only after extensive washing of the cells with the Ti-citrate-EDTA reagent described by Hudson and Morel (1989), as unwashed cells were found to bind a large amount of iron but in a time-independent manner indicative of rapid surface binding (Fig. 1). The amount of surface binding as compared to internalization was at least a 100-fold higher. While this surface binding of iron was not unexpected, it was initially dismissed as nonspecific and an experimental nuisance. However, upon further examination, it was found that the high level of surface binding persisted even when the iron in the growth media was presented in the form of highly stable EDTA or other chelates, which was inconsistent with weak nonspecific binding. Indeed, surface binding was not eliminated until the EDTA/Fe ratio approached 100:1, indicating that the stability of the Fe-surface interaction had to be greater than that of FeEDTA itself (Fig. 2). Measuring the surface binding from a solution of fixed [^55^Fe] (1 μM) as a function of [EDTA] and pH allowed an estimation of an effective surface-binding constant $K\;{'}_{eff}$ (reaction 1: see Supplementary Tables S1–S8 for detailed analysis). The values obtained were relatively constant ( $K\;{'}_{eff}$ = 3–29×10^21^ M^–1^) over a wide range (33–1500 μM) of excess EDTA, confirming uniform binding and reliability of the data set. At high pH (8.6–8.8), iron binding was very strong (log $K\;{'}_{eff}$ = 21–22) while at acidic pH (4.8) the binding constant was drastically reduced (log $K\;{'}_{eff}$ = 14), presumably due to protonation of the alginate carboxylate groups thought to be the major iron-binding moieties.

**Fig. 3.**
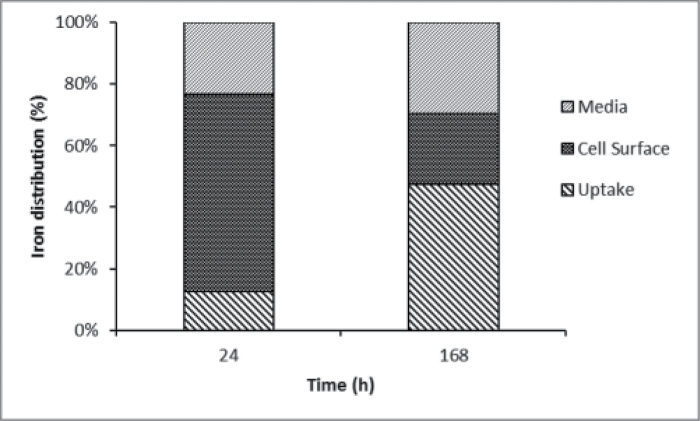
Distribution of ^55^Fe after exposure of *Ectocarpus* to a 30 μM solution of FeEDTA for 6h, followed by resuspension of cells in iron-free media for either 24 or 168h. Cell-surface iron represents the difference between samples before (total Fe) and after washing (internalized Fe) with the Ti-citrate-EDTA reagent. Data represent mean of three determinations.

**Fig. 4.**
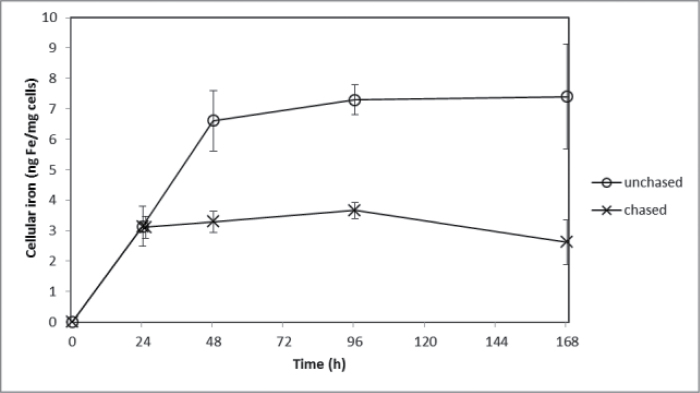
^55^Fe uptake (internalized Fe) after washing with the Ti reagent as a function of time by *E. siliculosus* treated with 30 μM ^55^FeEDTA or following a chase of 30 μM ^56^FeEDTA. The chase was added at the 24-h time point. Data are mean ± standard deviation of triplicate measurements.

**Fig. 5.**
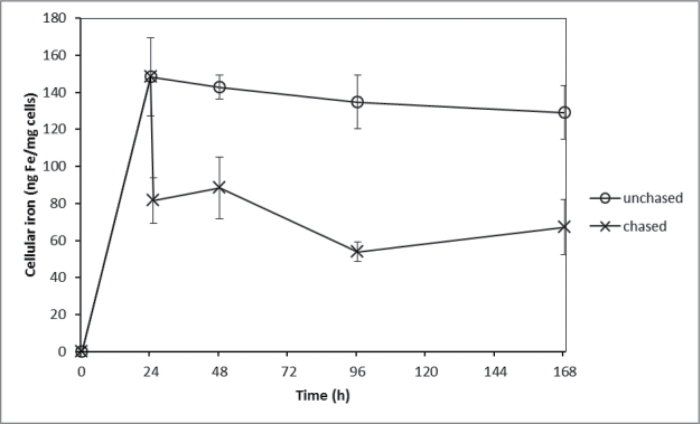
Total *E. siliculosus* cellular iron ^55^Fe uptake (i.e. not washed with the Ti reagent) as a function of time by *E. siliculosus* treated with 30 μM ^55^FeEDTA or following a chase of 30 μM ^56^FeEDTA. The chase was added at the 24-h time point. Data are mean ± standard deviation of triplicate measurements.

While it was clear that iron bound to the surface of *E. siliculosus* with high thermodynamic stability, its kinetic stability was undetermined. ^55^Fe pulse experiments were thus conducted to investigate the effect on uptake after initial binding at the cell surface. Pulse-labelling the cell surface with ^55^Fe followed by replacement of the media with ‘iron-free’ media (oligotrophic, open ocean water with a sub-nM concentration of iron) showed that approximately 50% of the surface-bound iron quickly re-entered solution while intracellular iron continued to increase with time (Fig. 3). However, ambiguity remained as to whether the increasing intracellular iron was derived directly from the surface-bound iron or from surface-bound iron that re-entered the solution before being taken up. The latter possibility was tested by a pulse-chase experiment in which an initial ^55^FeEDTA pulse was followed by a chase of ‘cold’ ^56^FeEDTA. Continued ^55^Fe uptake following the ^56^Fe chase would suggest that the Fe(III) taken up (internalized) was bound very tightly to the surface and did not re-enter the solution (and hence was unaffected by isotopic dilution). This, however, was not the case, as can be seen in Figs. 4 and 5 for Ti-washed and unwashed samples, respectively. The complete cessation of ^55^Fe uptake upon isotopic dilution implied that surface-bound iron is not internalized directly; rather it re-enters the bulk solution.

### Localization of iron on the surface

**Fig. 6.**
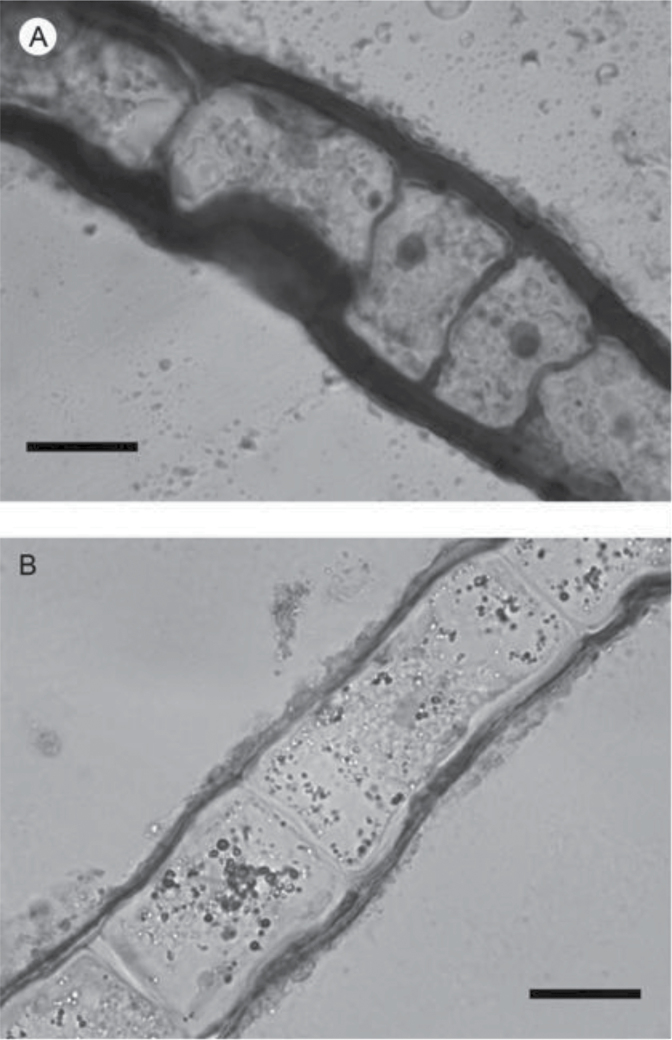
Light micrographs of *E. siliculosus* stained with the Perls-DAB procedure without (A) and with (B) Ti-citrate-EDTA washing: dark areas indicate high concentrations of iron. Bars, 10 μm.

**Fig. 7.**
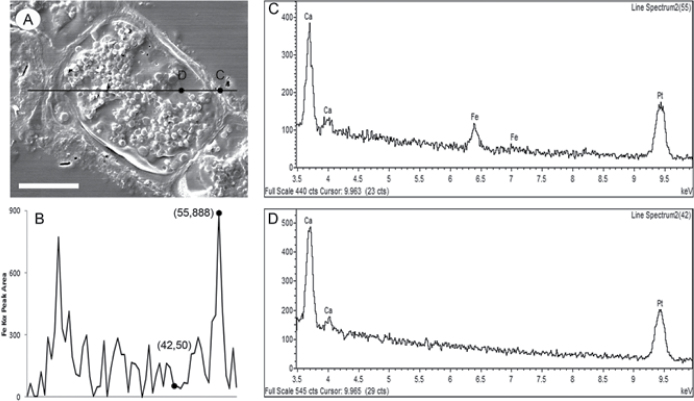
Scanning electron microscopy and energy-dispersive X-ray analysis of *E. siliculosus*. (A) Scanning electron micrograph of *E. siliculosus* without Ti-citrate-EDTA washing: black line depicts the transect for energy-dispersive X-ray analysis with two points. (C and D) selected for accompanied spectra (white bar, 10 μm). (B) Fe K_α_ peak areas of 60 points along the full transect, scaled to fit the line in A. (C and D) Energy-dispersive X-ray spectra of points C and D from the transect in A.

**Fig. 8.**
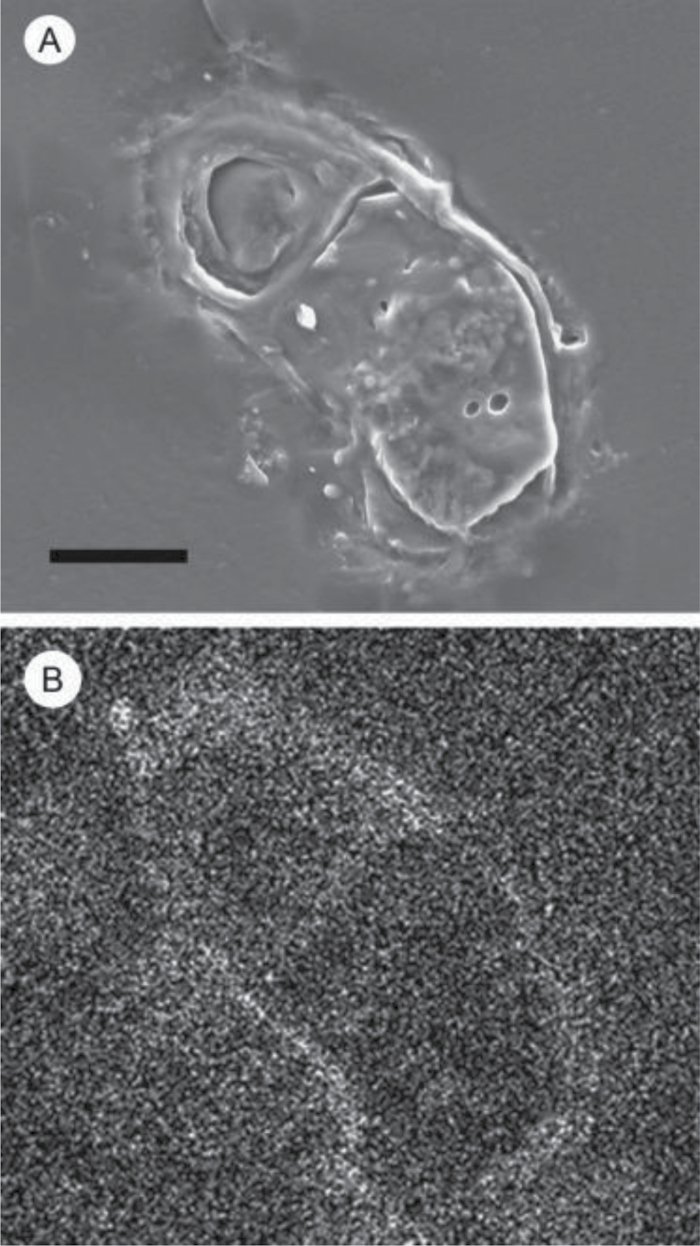
(A) Scanning electron microscopy of *E. siliculosus* not washed with T-citrate-EDTA (bar, 10 μm). (B) Energy-dispersive X-ray analysis of the same sample area as A: white pixilation represents areas with higher Fe K_α_ X-ray counts.

Microscopic analyses were performed to investigate the spatial distribution and uniformity of iron bound to the cell surface. The iron-specific stain Perls was lacking in intensity for optical microscopic analysis so the enhancing agent DAB was used in conjunction with Perls. This proved effective for detecting extracellular as well as intracellular iron (Fig. 6). It was clear that the majority of the iron in samples not washed with the Ti reagent was present on the surface with internal iron only visible in washed samples. The preponderance of surface-bound iron in unwashed samples was corroborated by energy-dispersive X-ray analysis (Fig. 7). Figure 8 depicts a scanning electron micrograph of an *E. siliculosus* cell alongside the corresponding energy-dispersive X-ray analysis of iron superimposed on the same field.

### Nature of the surface-bound iron

**Fig. 9.**
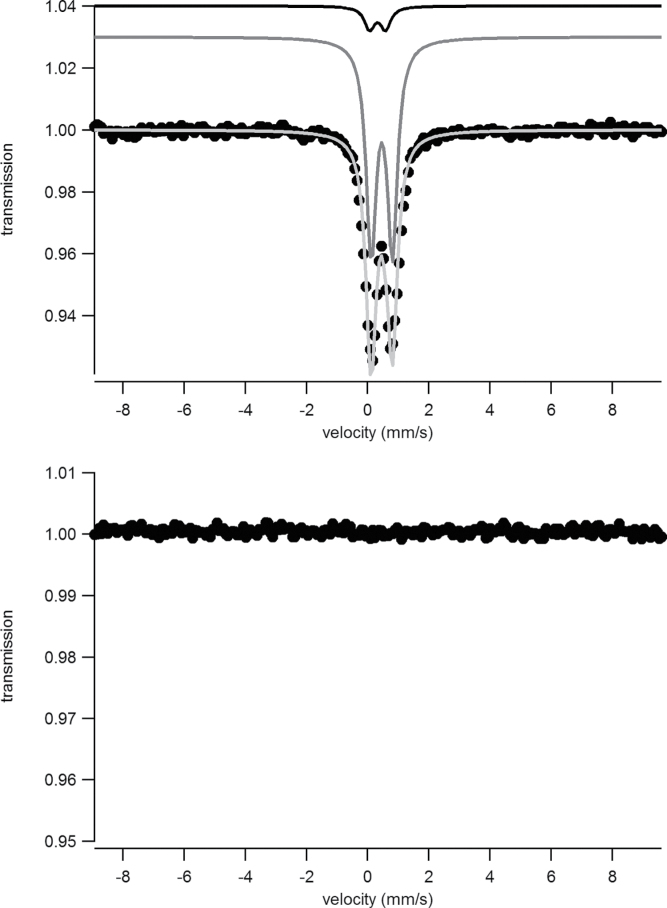
TMS at 77 K of samples of *Ectocarpus* grown before (A) and after (B) washing with Ti-citrate-EDTA reagent: dots, experimental data; dark grey line, Fe-carboxylate species; black line, unknown species; light grey line, the fit to the data using the parameters given in Table 1.

**Fig. 10.**
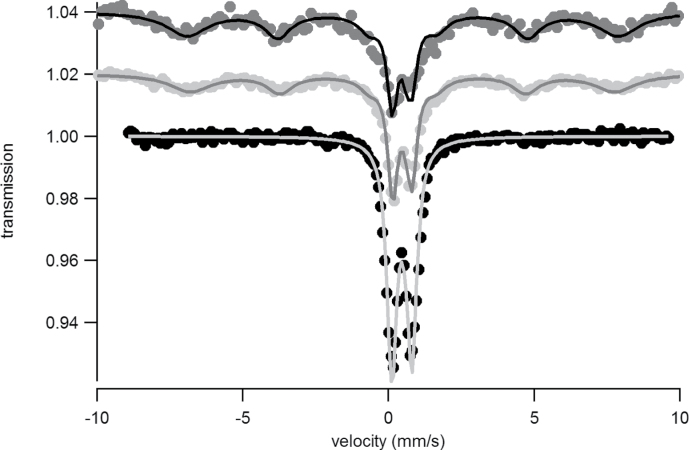
TMS at different temperatures of a sample of *Ectocarpus* grown before washing with Ti-citrate-EDTA reagent: black dots, 1.8 K; light grey dots, 4.2 K; dark grey dots, 77 K; solid lines, the fit to the data using the parameters given in Table 1.

TMS and XAS were used to determine more precise details of the surface iron and its surrounding ligands. The TMS spectra of a sample of *Ectocarpus* grown for 48h exposed to 30 μM ^57^FeEDTA and either washed with Ti-citrate reagent or unwashed can be seen in Fig. 9B and A, respectively. Essentially no signal was detectable in the washed sample indicating that, as expected (Böttger *et al.*, 2012), the amount of iron internalized after short-term incubation was below the TMS detection limit. Therefore the entire signal seen in the unwashed sample represented external or surface-bound iron. The data for the unwashed sample could be fitted (Fig. 9A) with two doublets exhibiting the parameters shown in Table 1. The main component (c2a + c2b) had parameters which are typical for polymeric Fe(III) octahedrally coordinated to primarily oxygen ligands and fitted into the range observed for carboxylate Fe(III) model complexes (Dziobkowski *et al.*, 1981). The second and very minor component (~10% of total area) exhibited parameters that belong to an as-yet unknown species. The parameters obtained for the major component were similar to but distinct from (Δδ = 0.05mm s^–1^ and different relaxation behaviour, as will be discussed further) those derived earlier (Böttger *et al.*, 2012) for the major component of the internalized iron seen upon long-term exposure. The most significant difference was in the blocking temperature (Mørup, 2011) obtained from the temperature dependence of the TMS spectrum (Fig. 10), which was above 4.2 K for the surface-bound iron as compared to below 1.8 K for that internalized. In the case of mineral species, this behaviour would normally suggest either a more crystalline structure and/or larger particle sizes.

**Table 1.** Parameters used to fit the TMS of sample of *Ectocarpus* grown for 48h on 30 μM ^57^FeEDTA at various temperatures Component c2b is the split part of c2a (Mørup, 2011).

Temperature (K)	Component	δ (mm s^–1^)	ΔEq (mm s^–1^)	B_hf_	Relative area (%)
77	c1	0.32	0.52	–	~10
	c2a	0.46	0.69	–	~90
	c2b	–	–	–	–
4.2	c1	0.34	0.52	–	~10
	c2a	0.49	0.65	–	~35
	c2b	0.49	0	44.7	~55
1.8	c1	0.34	0.52	–	~12
	c2a	0.49	0.65	–	~20
	c2b	0.49	0	45.4	~68

**Fig. 11.**
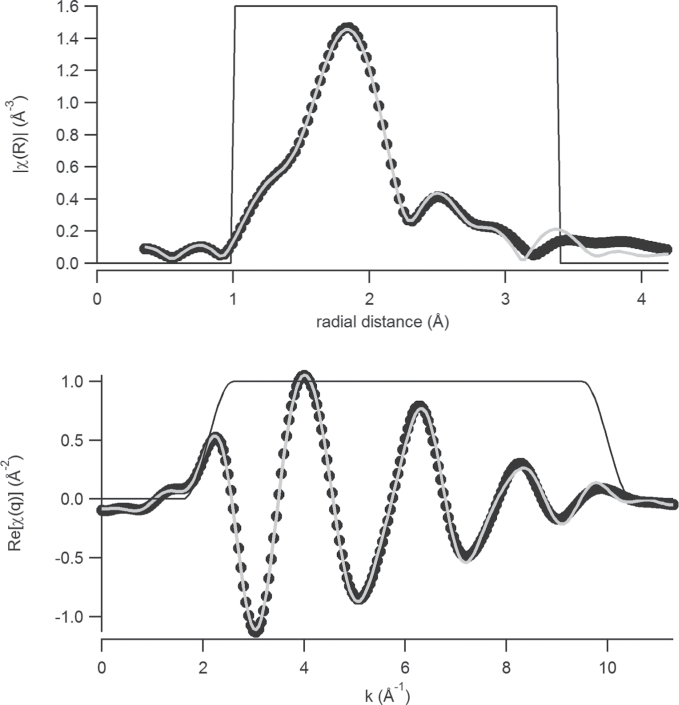
Spectrum of extended X-ray analysis of fine structure in R-space (top) and q-space (bottom). Black dots, experimental data; light grey line, the fit; black line, Hanning-type windows.

In addition to TMS, this study employed XAS to probe the chemical nature of the external iron. The EXAFS spectrum for an unwashed sample of *Ectocarpus* is shown in Fig. 11. Based on the above Mössbauer data, the presence of a superparamagnetic polymeric Fe(III)-oxo system was indicated. Within this constraint, various models were tested. Models for species were constructed and coordinates were obtained after molecular mechanics energy minimization using the program Chem 3D. These coordinates were then used as input to the FEFF program to obtain the scattering paths. The best fits came from a hydroxo-carboxylato model (see Supplementary Model). The final fit of the EXAFS spectrum (Fig. 11) to the model was very good, where there were 3.4±0.8 bridging carboxylate groups Fe-O-C-[COR] present with bond distances of Fe-O of 1.99 Å (n = 4.2±0.6), Fe-[O]-C of 2.68 Å (n=3.3±1.2), Fe-[O-C]-O of 2.90 Å (*n* = 2.6±0.9) and 0.9±0.4 OH groups with an Fe-O bond distance of 1.81 Å. Overall, the EXAFS fit data were completely consistent with that obtained by TMS.

## Discussion

Until recently, adsorption of iron to the cell surface of marine algae has been viewed as an experimental artefact that complicates uptake studies. While it is indeed necessary to remove this signal when quantifying uptake, the potential biological significance of this surface-bound iron has generally been ignored. Such was the case in the previous study of *E. siliculosus* (Böttger *et al.*, 2012). However in the process of reexamining the data, the current study group was intrigued by the fact that a high level of surface binding was occurring despite the fact that all the iron present in the media was in the form of the very stable FeEDTA chelate. Indeed surface binding persisted even when an excess of EDTA was present. Thus it appeared that the binding of iron to the surface of *E. siliculosus* is far stronger than that expected for a weak nonspecific electrostatic interaction and thus might have some biological significance. This possibility was supported by the estimate of the effective surface-binding constant ( $K\;{'}_{eff}$ ) for Fe(III)' of 3–29×10^21^ M^–1^, which was comparable to the affinity of EDTA' for Fe(III)' at pH 8.7 (Supplementary Table S8). The magnitude of the drop in affinity with decreasing pH was also comparable to that observed for EDTA, which is a polycarboxylic acid and was thus consistent with the binding of iron to the cell surface of *Ectocarpus* primarily by the carboxylate groups found in the alginates, sulphated polysaccharides, and pectins, which are the major cell-wall components in this class of organism. Histological staining and energy-dispersive X-ray analysis of both environmental and laboratory-derived samples of *E. siliculosus* confirmed the spatial uniformity and primarily surface binding of iron, as would be expected of a constitutive scaffold such as the cell wall. While the numerous subgroups and variable side chains of these components presented a challenge for the identification of the specific iron-binding site(s), this study used a combination of TMS and EXAFS spectroscopic studies to enable the determination of iron oxidation state, geometry, and ligation in the first and second coordination spheres. The results are entirely consistent with octahedral hydroxo-carboxylato ligation of the iron.

**Fig. 12.**
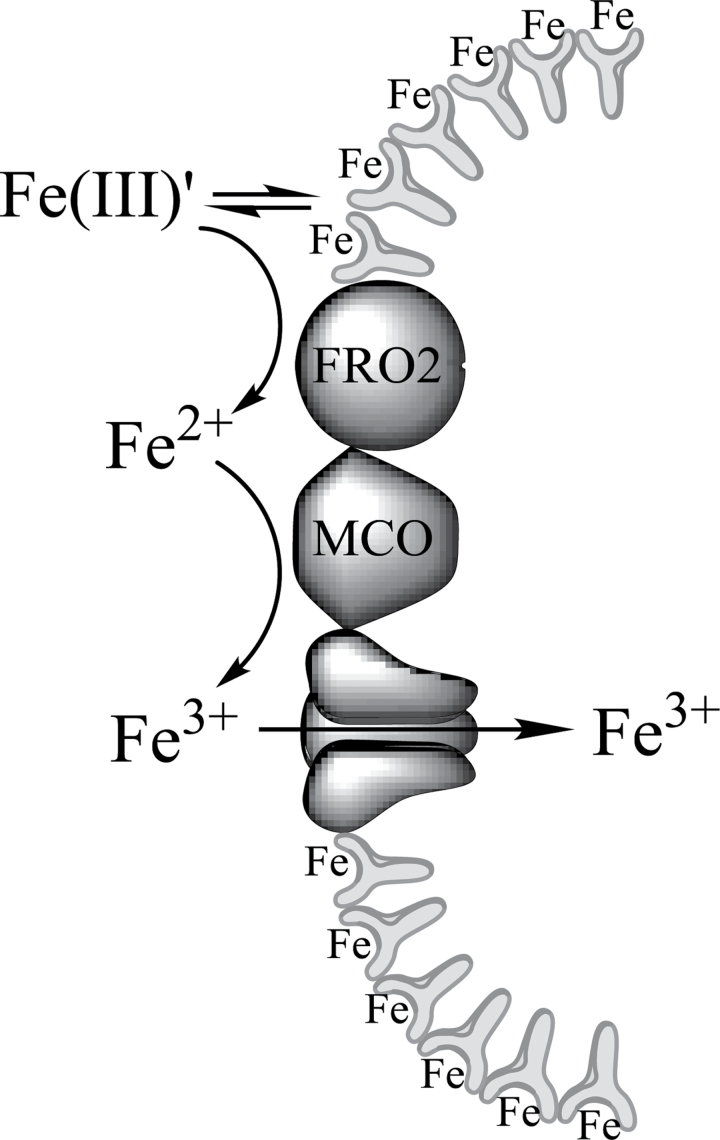
Proposed mechanism for iron uptake in *Ectocarpus.* Free ferric ion in all its soluble forms (depicted as Fe(III)′) is in equilibrium between the solution phase and the cell surface. The solution-phase Fe(III) is reduced to Fe(II) by a cell-surface reductase homologous to the FRO2 protein of *Arabidopsis*. The resulting Fe(II) is then reoxidized by a multicopper oxidase (MCO) coupled to an Fe(III) permease for final transport into the cell.

Finally, the question remains: what is the biological significance (if any) of this strong surface binding of iron? While open-ocean-dissolved iron concentrations are virtually always extremely low (sub-nM), concentrations of this element in coastal regimes are far more variable and range from 0.1 to 300nM (Chase *et al.*, 2002). Thus organisms in coastal ecosystems have to contend with an inherently different set of acquisition problems as compared with organisms in oligotrophic open ocean environments. In particular, iron concentrations are subject to considerable variation depending on a variety of climatic conditions. Thus coastal organisms can be subjected to either very low or very high concentrations of iron in their environment. Surface binding of iron could serve as a metal ion buffer to assure organisms like *Ectocarpus* of an adequate supply of iron for metabolic needs in the face of widely varying external concentrations of the element. In times of plenty the equilibrium would strongly favour iron bound to the surface, while in times of deprivation the equilibrium would shift to dissociation of the surface-bound iron into solution. This study’s overall proposed mechanism for iron uptake in *Ectocarpus*, based on the data presented here and in Böttger *et al*. (2012), is shown in Fig. 12.

Since the data most closely support a model where iron uptake is from the solution phase rather than directly from the cell surface, such equilibria would assure an adequate solution iron concentration for efficient uptake only if the solution-phase iron derived from the surface was not free to diffuse away. However, in non-uniseriate plants, the cell-wall space (apoplast) between cells is interconnected and serves as a continuous matrix through which surface-bound nutrients could experience highly restricted diffusion despite the fact that it does not serve an obvious transport function. Maintaining a diverse cast of apoplastic polysaccharides with anionic ligands of considerable strength could be a strategy employed by *E. siliculosus* and likely by many other marine macroalgae to buffer the widely fluctuating coastal iron concentrations. Such a mechanism could be biologically important and allow these organisms to be competitive particularly in high-iron environments. Possible ecological evidence for this mechanism comes from the dramatic iron-induced phase shifts seen around some of the reefs on the Line Islands where corals dominate under natural oligotrophic conditions while macro and turf algae dominate near the site of recent shipwrecks releasing large amounts of iron into the shallow-water marine environment (Kelly *et al.*, 2012).

## SUPPLEMENTARY MATERIAL

Supplementary data are available at *JXB* online.

Supplementary Material S1. Surface iron-binding constant and equilibrium studies.

Supplementary Table S1. Experimental data.

Supplementary Table S2. Important parameters calculated for the reaction between cell and FeEDTA.

Supplementary Table S3: Ringbom coefficients1 calculated for the equilibrium between Fe(III) and EDTA and equilibrium constant *β′*.

Supplementary Table S4. Binding constants (*K′*, *K*. and ${\text{Fe}}_{\text{T}}^{3+}$ ) for the reaction between cell and FeEDTA.

Supplementary Table S5. Protonation constants of EDTA.

Supplementary Table S6. log *K* values for FeEDTA.

Supplementary Table S7. log *K* values for Fe(III).

Supplementary Table S8. Calculated Fe(III) affinity constants of iron for cell ( ${\text{Fe}}_{\text{T}}^{3+}$ ) and EDTA (*β′*).

Supplementary Model. Model used to calculate scattering paths for extended X-ray analysis of fine structure.

Supplementary Data
