# Supplementary material for: Surface-bound iron: a metal ion buffer in the marine brown alga Ectocarpus siliculosus?
Source: J Exp Bot. 2013 Dec 24;65(2):585–94. doi: 10.1093/jxb/ert406 (PMC3904714; doi:10.1093/jxb/ert406)
Supplement: Supplementary Data [file supp_65_2_585__index.html]

Surface-bound iron: a metal ion buffer in the marine brown alga Ectocarpus siliculosus? — Surface-bound iron: a metal ion buffer in the marine brown alga Ectocarpus siliculosus? — Supplementary Data 

# Surface-bound iron: a metal ion buffer in the marine brown alga *Ectocarpus siliculosus*?

## Supplementary Data

Data files

**Files in this Data Supplement:**

- Supplementary Data - Supplementary Data
