# Supplementary material for: Surface-bound iron: a metal ion buffer in the marine brown alga Ectocarpus siliculosus?
Source: J Exp Bot. 2013 Dec 24;65(2):585–94. doi: 10.1093/jxb/ert406 (PMC3904714; doi:10.1093/jxb/ert406)

Supplementary Material

Surface Bound Iron: A metal ion Buffer in the Marine Brown Alga *Ectocarpus siliculosus*?

Eric P. Miller[1], Lars H. Böttger[2,5], Aruna J. Weerasinghe[3], Alvin L. Crumbliss[3], Berthold F. Matzanke[2], Frithjof C. Küpper[4], and Carl J. Carrano[1*]

Surface Iron Binding Constant and Equilibrium Studies.

Overall approach. Cell surface affinity for Fe^{3+} is quantified by defining a binding constant (K'_{eff}) which represents the binding of $[Fe_T^{3+}]_{eq}$ species with cells according to reaction (1). In this equation, $[Fe_T^{3+}]_{eq}$ represents all soluble forms of Fe^{3+} in the equilibrium. Reaction (2), which represents the experimentally observed reaction between cells and FeEDTA, was considered to obtain equation (6). K' and K are equilibrium constants for reactions (2) and (7) respectively. Ringbom coefficients[1] for Fe^{3+}, EDTA, and FeEDTA calculated from known equilibrium constants were used to obtain stability constants. The calculated (K'_{eff}) shows a strong binding of Fe^{3+} with cells in a pH dependent manner.

Assumptions. Three assumptions were made in treating the experimental data.

1. The only species that binds to the cell surface is naked iron, $[Fe_T^{3+}]_{eq}\{Fe(H_2O)_6^{3+} + Fe(OH)^{2+} + Fe(OH)_2^{+} + Fe_2(OH)_2^{4+}\}$.
2. Neither EDTA nor FeEDTA binds to the cell surface.
3. Weight of the iron bound to the cell surface is considered as FeCell.

Calculation Method. Our objective is to determine the affinity of $[Fe_T^{3+}]_{eq}$ for the cell surface by calculating the equilibrium constant K'_{eff} for reaction (1) at a specific pH.

$$Cell + [Fe_T^{3+}]_{eq} \leftrightarrows FeCell \qquad (1) \qquad K'_{eff}$$

We experimentally observe the following reaction (2) between Cell and $FeEDTA'$.

$$Cell + FeEDTA' \leftrightarrows FeCell + EDTA' \quad (2) \quad K'$$

$$EDTA' = Total\ species\ of\ EDTA\ in\ equilibrium$$
$$= EDTA^{4-} + HEDTA^{3-} + H_2EDTA^{2-} + H_3EDTA^{-} + H_4EDTA + H_5EDTA^{+}$$

$$FeEDTA' = Total\ species\ of\ FeEDTA\ in\ equilibrium$$
$$= FeEDTA^{-} + FeHEDTA + Fe(OH)EDTA^{2-}$$

$$[Fe_T^{3+}]_{eq} = Total\ species\ of\ free\ Fe^{3+} in\ equilibrium$$
$$= Fe(H_2O)_6^{3+} + Fe(OH)^{2+} + Fe(OH)_2^{+} + Fe_2(OH)_2^{4+}$$

$$K' = \frac{[FeCell][EDTA]'}{[Cell][FeEDTA]'} \quad (3)$$

$$K' = \frac{[FeCell]}{[Cell][Fe_T^{3+}]_{eq}} X \frac{[Fe_T^{3+}]_{eq}[EDTA]'}{[FeEDTA]'} \quad (4)$$

$$\beta' = \frac{[FeEDTA]'}{[Fe_T^{3+}]_{eq}[EDTA]'} \quad (5)$$

$$K' = \frac{K'_{eff}}{\beta'} \quad (6)$$

K' in equations (3) – (6) can be corrected for EDTA and FeEDTA speciation using Ringbom coefficients[1] to obtain K (equation (10)) for reaction (7). FeCell and Cell represent all protonated forms.

$$Cell + FeEDTA^{-} \leftrightarrows FeCell + EDTA^{4-} \quad (7) \quad K$$

$$K = \frac{[FeCell][EDTA^{4-}]}{[Cell][FeEDTA^{-}]} \quad (8)$$

$$K' = \frac{[FeCell][EDTA^{4-}]\alpha_{EDTA}}{[Cell][FeEDTA^{-}]\alpha_{EDTA}} \quad (9)$$

$$K = \frac{K'\alpha_{FeEDTA}}{\alpha_{EDTA}} \quad (10)$$

Equations (3) – (10) were obtained considering the equilibrium between the Cell and $FeEDTA'$ (reaction (2)) where K' is the equilibrium constant for Reaction (2) at a specific pH. K'_{eff} is the effective binding constant for the cell and $[Fe_T^{3+}]_{eq}$ according to equilibrium reaction (1).

Iron uptake by cells was monitored in the presence of varying concentrations of EDTA at a constant iron concentration. The amount of cells used in each experiment was also varied over a

wide range (10 – 18.6 mg). All relevant experimental data are summarized in Table S1. Calculated equilibrium concentrations are summarized in Table S2.

Ringbom coefficients for iron, EDTA and FeEDTA were calculated using equations (11) – (24) (Table S3). β' obtained using Ringbom coefficients are also presented in Table S3. Affinity constants calculated using equilibrium concentrations and Ringbom coefficients are given in Table S4.

$$EDTA + nH \quad \leftrightarrows \quad H_nEDTA \quad (11)$$

$$\beta_n^H = \frac{[H_nEDTA]}{[EDTA][H]^n} \quad (12)$$

$$[EDTA]' = \alpha_{EDTA}\,[EDTA^{4-}] \quad (13)$$

$$\alpha_{EDTA} = 1 + \beta_1^H[H] + \beta_2^H[H]^2 \ldots\ldots\ldots + \beta_5^H[H]^5 \quad (14)$$

Successive log $\beta_n^H(n = 1 - 5)$ values (10.19, 16.32, 19.01, 21.01, and 22.51) were calculated from protonation constants for EDTA (Table S5).[2]

$$EDTA^{4-} + Fe^{3+} \quad \leftrightarrows \quad FeEDTA^{-} \quad (15) \quad \beta_{110}$$

$$FeEDTA^{-} + H^{+} \quad \leftrightarrows \quad FeHEDTA \quad (16) \quad K_1$$

$$Fe(OH)EDTA^{2-} + H^{+} \quad \leftrightarrows \quad FeEDTA^{-} \quad (17) \quad K_2$$

$$\beta_{110}^{FeEDTA} = \frac{[EDTA^{-}]}{[Fe_{aq}^{3+}][EDTA^{4-}]} \quad (18)$$

$$[FeEDTA]' = \alpha_{FeEDTA}\,[FeEDTA^{-}] \quad (19)$$

$$\alpha_{FeEDTA} = 1 + K_1[H^{+}] + 1/(K_2[H^{+}]) \quad (20)$$

Log β values used in the calculation of α_{FeEDTA} were ($\beta_{110} = 25.1,\ K_1 = 1.3, K_2 = 7.37$) for FeEDTA (Table S6).[2]

$$Fe(H_2O)_6^{3+} + OH^{-} \quad \rightleftarrows \quad Fe(OH)^{2+} \quad (21) \quad K_1$$

$$Fe(H_2O)_6^{3+} + 2OH^{-} \quad \rightleftarrows \quad Fe(OH)_2^{2+} \quad (22) \quad K_2$$

$$[Fe_T^{3+}]_{eq} = [Fe(H_2O)_6^{3+}]\ X\ \alpha_{Fe(OH)} \quad (23)$$

$$\alpha_{Fe(OH)} = 1 + K_1\,[OH] + K_2[OH]^2 \quad (24)$$

Formation of $Fe_2(OH)_2^{4+}$ depends on the concentration of Fe^{3+}. In most calculations, the concentration of Fe^{3+} is less than $10^{-3.7}$M. Therefore, $\alpha_{Fe(OH)}$ can be calculated neglecting $Fe_2(OH)_2^{4+}$. Log K_1 and K_2 are 11.0 and 21.7 respectively (Table S7).

Table S1: Experimental data[a]

Expt.	pH	$[Fe]_{Init}$ / µM	$[EDTA]_{Init}$ / µM	$[Cell]_{Init}$/ mg	Fe uptake ng Fe/mg cell
1	8.7	30	33	11.2	998.8865
2	8.7	30	33	13.9	444.1926
3	8.7	30	33	11.2	602.3859
4	8.8	30	300	13.1	224.0947
5	8.8	30	300	18.6	179.4181
6	8.8	30	300	10.6	236.3291
7	8.6	30	1500	12.8	42.32842
8	8.6	30	1500	10.5	50.79342
9	8.6	30	1500	11.6	42.99006
10	4.8	30	3000	14.5	0.564329
11	4.8	30	3000	10	0.760243
12	4.8	30	3000	11.3	1.042555

[a] $[Fe]_{Init}$= Initial amount of iron added. $[EDTA]_{Init}$ = Initial amount of EDTA added. $[Cell]_{Init}$= Initial weight of cells.

Table S2: Important parameters calculated for the reaction between Cell and FeEDTA[a]

Expt.	$[FeCell]_{eq}$ /mg	$[Cell]_{eq}$/ mg	$[EDTA]'_{eq}$ /µM	$[FeEDTA]'_{eq}$ /µM
1	0.01118	11.1888	13.018	19.98
2	0.00617	13.8938	8.53	24.47
3	0.00674	11.1932	9.04	23.96
4	0.00293	13.0970	272.63	27.37
5	0.00333	18.5966	272.98	27.01
6	0.00250	10.5975	272.24	27.76
7	0.00054	12.7994	1470.49	29.51
8	0.00053	10.4995	1470.48	29.52
9	0.00049	11.5995	1470.15	29.55
10	8.18×10^{-6}	14.4999	2970.007	29.99
11	7.60×10^{-6}	9.9999	2970.007	29.99
12	1.18×10^{-5}	11.2999	2970.011	29.99

[a] $[FeCell]_{eq}$ = Weight of FeCell in the equilibrium. $[Cell]_{eq}$ = Weight of cell in the equilibrium. $[EDTA]'_{eq}$ = Concentration of total EDTA speciation in the equilibrium; see statement following reaction (2). $[FeEDTA]'_{eq}$ = Concentration of total FeEDTA in the equilibrium; see statement following reaction (2). $[Fe_T^{3+}]_{eq}$= Concentration of total free iron in the equilibrium; see statement following reaction (2).

.

Table S3: Ringbom coefficients[1] calculated for the equilibrium between Fe^{3+} and EDTA and equilibrium constant $\boldsymbol{\beta'}$

pH	$\boldsymbol{\alpha_{EDTA}}$	$\boldsymbol{\alpha_{FeEDTA}}$	$\boldsymbol{\alpha_{Fe(OH)}}$	$\boldsymbol{\beta'}$ [a]
8.8	25.599	27.915	1.99×10^{11}	8.81×10^{24}
8.7	31.986	22.379	1.25×10^{11}	1.37×10^{25}
8.6	40.036	17.982	7.94×10^{10}	5.65×10^{24}
4.8	5534346.12	1.003	2.06×10^{3}	2.28×10^{18}

[a] See eqn (5).

Table S4: Binding constants ($K', K,$ and K'_{eff}) for the reaction between cell and FeEDTA[a]

Expt.	$\boldsymbol{K'}$	Average $\boldsymbol{K'}$	$\boldsymbol{K}$	Average $\boldsymbol{K}$	$\boldsymbol{K'_{eff}}$	Average $\boldsymbol{K'_{eff}}$
1	6.52×10^{-4}	$3.4 \pm 2.7 \times 10^{-4}$	4.55×10^{-4}	$2.4 \pm 1.8 \times 10^{-4}$	5.74×10^{21}	3.03×10^{21}
2	1.55×10^{-4}		1.08×10^{-4}		1.36×10^{21}	
3	2.27×10^{-4}		1.59×10^{-4}		2.00×10^{21}	
4	2.23×10^{-3}	$2.1 \pm 0.3 \times 10^{-3}$	2.43×10^{-3}	$2.3 \pm 0.3 \times 10^{-3}$	3.06×10^{22}	2.91×10^{22}
5	1.81×10^{-3}		1.97×10^{-3}		2.48×10^{22}	
6	2.32×10^{-3}		2.52×10^{-3}		3.18×10^{22}	
7	2.11×10^{-3}	$2.3 \pm 0.2 \times 10^{-3}$	9.47×10^{-4}	$1.0 \pm 0.1 \times 10^{-3}$	1.19×10^{22}	1.27×10^{22}
8	2.53×10^{-3}		1.14×10^{-3}		1.43×10^{22}	
9	2.13×10^{-3}		9.60×10^{-4}		1.21×10^{22}	
10	5.59×10^{-5}	$7.8 \pm 2.4 \times 10^{-5}$	1.01×10^{-11}	$1.4 \pm 0.4 \times 10^{-11}$	1.27×10^{14}	1.78×10^{14}
11	7.53×10^{-5}		1.36×10^{-11}		1.72×10^{14}	
12	1.03×10^{-4}		1.87×10^{-11}		2.35×10^{14}	

[a] $\boldsymbol{K'}$ see eqn (9). $\boldsymbol{K}$ see eqn (8). $\boldsymbol{K'_{eff}}$ see eqns (1) and (6).

Sample calculation (Table S2)

30 µM Fe = 33.5 µg of Fe

Iron bound to cells (Expt 1), $[\boldsymbol{FeCell}]_{\boldsymbol{eq}}$ = (998.8865 ng/mg) x (11.2 mg Cells) = 11.1875 µg = 10.0186 µM = **0.01118 mg**

Therefore Fe bound to EDTA (Expt 1), $[\boldsymbol{FeEDTA}]'_{\boldsymbol{eq}}$ = 33.5 µg -11.1875 µg =

22.31248 µg = **19.9813 µM**

Cells in the equilibrium (Expt 1), $[\boldsymbol{Cell}]_{\boldsymbol{eq}}$ = 11.2 mg – 0.01118 mg = **11.188 mg**

EDTA in the equilibrium (Expt 1), $[\boldsymbol{EDTA}]'_{\boldsymbol{eq}}$ = 33.0 µM – 19.9813 µM = **13.018 µM**

Table S5: Protonation constants of EDTA (μ = 0.1, T = 20 °C)[2]

EDTA Equilibrium	Log K
$[HEDTA^{3-}]/[EDTA^{4-}][H^+]$	10.19
$[H_2EDTA^{2-}]/[HEDTA^{3-}][H^+]$	6.13
$[H_3EDTA^-]/[H_2EDTA^{2-}][H^+]$	2.69
$[H_4EDTA]/[H_3EDTA^-][H^+]$	2.0
$[H_5EDTA^+]/[H_4EDTA][H^+]$	1.5

Table S6: Log K values for FeEDTA (μ = 0.1, T = 20 °C)[2]

FeEDTA Equilibrium	Log K or β
$[FeEDTA^-]/[Fe^{3+}][EDTA^{4-}]$	25.1
$[FeHEDTA]/[FeEDTA^-][H^+]$	1.3
$[FeEDTA^-]/[Fe(OH)EDTA^{2-}][H^+]$	7.39

Table S7: Log K values for FeEDTA (μ = 0.1, T = 20 °C)[1]

Fe^{3+} Equilibrium	Log K
$[Fe(OH)^{2+}]/[Fe(H_2O)_6^{3+}][OH^-]$	11.0
$[Fe(OH)_2^+]/]/[Fe(H_2O)_6^{3+}][OH^-]^2$	21.7

Calculated Fe^{3+} affinity constants of iron for Cell (K'_{eff}) and EDTA (β') are given in Table S8 for comparison purposes.

Table S8: Calculated Fe^{3+} affinity constants of iron for Cell (K'_{eff}) and EDTA (β') [a]

<table>
<tr><th>Expt.</th><th>K'_{eff}/ M^{-1}</th><th>β' / M^{-1}</th></tr>
<tr><td>1</td><td rowspan="3">3.03 x 10^{21}</td><td rowspan="3">8.81 x 10^{24}</td></tr>
<tr><td>2</td></tr>
<tr><td>3</td></tr>
<tr><td>4</td><td rowspan="3">2.91 x 10^{22}</td><td rowspan="3">1.37 x 10^{25}</td></tr>
<tr><td>5</td></tr>
<tr><td>6</td></tr>
<tr><td>7</td><td rowspan="3">1.27 x 10^{22}</td><td rowspan="3">5.65 x 10^{24}</td></tr>
<tr><td>8</td></tr>
<tr><td>9</td></tr>
<tr><td>10</td><td rowspan="3">1.78 x 10^{14}</td><td rowspan="3">2.28 x 10^{18}</td></tr>
<tr><td>11</td></tr>
<tr><td>12</td></tr>
</table>

[a] K'_{eff} see eqns (1) and (6). β' see eqn (5).

References

1. A. Ringbom, Complexation in Analytical Chemistry: *A Guide for the Critical Selection of Analytical Methods Based on Complexation Reactions*: Interscience: New York, **1963**.

2. A. E. Martell, R. M. Smith, *Critical Stability Constants*: Plenum: New York, **1974**.

Model used to calculate scattering paths for EXAFS.

path	cw ratio	deg	n_{leg}	r_{eff} (Å)	atom
1	100.000	5.000	2	1.8147	O
2	19.214	1.000	2	1.8487	O
5	8.766	1.000	2	2.4615	C
7	1.152	2.000	3	2.5425	C
13	6.913	1.000	2	2.9016	O

Distances from Fe given in column 5.

Distances from model (see below):

Fe-C (bidentate carboxylate) 2.5 Å
Fe-OH 1.8 Å
Fe-C (bridging carboxylate) 2.7 Å
Fe-$O_{carboxylate}$ 2.9 Å

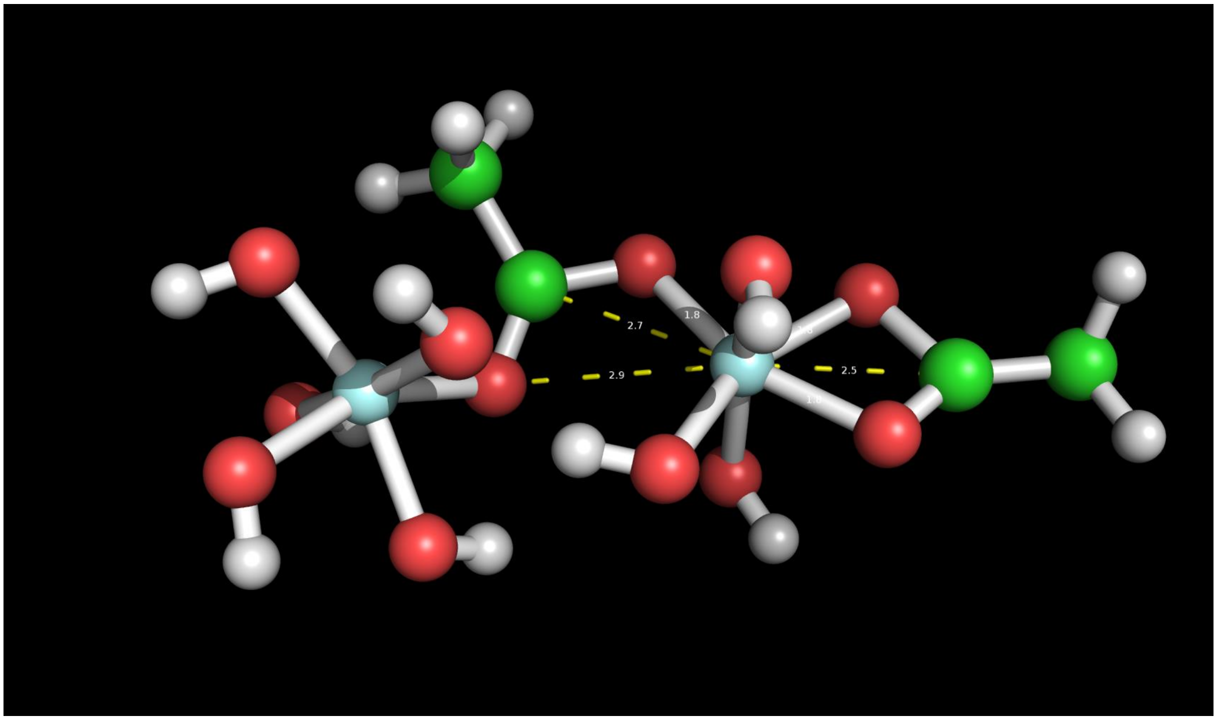

Supplement: Supplementary Data [file supp_ert406_jexbot109884_file001.pdf]
